# Medical residents and attending physicians’ perceptions of feedback and teaching in the United States: a qualitative study

**DOI:** 10.3352/jeehp.2022.19.9

**Published:** 2022-04-26

**Authors:** Madeleine Matthiesen, Michael S. Kelly, Kristina Dzara, Arabella Simpkin Begin

**Affiliations:** 1 Department of Medicine, Massachusetts General Hospital, Boston, MA, USA; 2 Department of Pediatrics, Massachusetts General Hospital, Boston, MA, USA; 3 Harvard Medical School, Boston, MA, USA; 4 Department of Biomedical Informatics and Medical Education and Center for Leadership and Innovation in Medical Education, University of Washington School of Medicine, Seattle, WA, USA; 5 Department of Medical Education, University of Washington School of Medicine, Seattle, WA, USA; 6 Center for Leadership and Innovation in Medical Education, University of Washington School of Medicine, Seattle, WA, USA; Hallym University, Korea

**Keywords:** Feedback, Internship and residency, Teaching, Qualitative research

## Abstract

**Purpose:**

Residents and attendings agree on the importance of feedback to resident education. However, while faculty report providing frequent feedback, residents often do not perceive receiving it, particularly in the context of teaching. Given the nuanced differences between feedback and teaching, we aimed to explore resident and attending perceptions of feedback and teaching in the clinical setting.

**Methods:**

We conducted a qualitative study of internal medicine residents and attendings from December 2018 through March 2019 at the Massachusetts General Hospital to investigate perceptions of feedback in the inpatient clinical setting. Residents and faculty were recruited to participate in focus groups. Data were analyzed using thematic analysis to explore perspectives and barriers to feedback provision and identification.

**Results:**

Five focus groups included 33 total participants in 3 attending (n=20) and 2 resident (n=13) groups. Thematic analysis of focus group transcripts identified 7 themes which organized into 3 thematic categories: (1) disentangling feedback and teaching, (2) delivering high-quality feedback, and (3) experiencing feedback in the group setting. Residents and attendings highlighted important themes in discriminating feedback from teaching. They indicated that while feedback is reactive in response to an action or behavior, teaching is proactive and oriented toward future endeavors.

**Conclusion:**

Confusion between the critical concepts of teaching and feedback may be minimized by allowing them to each have their intended impact, either in response to prior events or aimed toward those yet to take place.

## Introduction

### Background

Feedback is a critical component of resident education [1-3], providing information related to a learner’s performance intended to guide future thinking and behavior [1]. Beyond its practical necessity to correct mistakes and help learners grow, feedback improves the quality and quantity of learning, learner satisfaction, and even the learning environment [2,3]. As residency programs have shifted towards competency-based education, feedback has become even more critical. The Accreditation Council for Graduate Medical Education (ACGME) now includes a resident’s ability to learn and improve via feedback among its milestone competencies [4].

Although residents and faculty agree on the importance of feedback [5,6], they often disagree on the frequency of feedback in clinical scenarios [7]. We previously conducted a national survey of internal medicine residents demonstrating that residents may have difficulty identifying feedback in the context of teaching [7]. Whereas feedback provides information related to a learner’s performance intended to improve future work [2], teaching relates to providing information to learners based on an identified gap or need [8]. Given that attendings and residents disagree on the frequency but not the importance of feedback on clinical teams, better understanding the confusion around feedback and teaching may represent an opportunity to enhance clinical learning.

### Objectives

In this study, we held focus groups to explore resident and attending perceptions of the nuanced differences between feedback and teaching. We anticipated that participants would describe both the importance of differentiating feedback and teaching in clinical learning environments and identify specific language, situations, and perspectives used to assist them in this endeavor.

## Methods

### Ethics statement

This study was approved by the Institutional Review Board of the Massachusetts General Hospital (IRB#2018P001900). Subjects provided informed consent.

### Research team and reflexivity

#### Personal characteristics

The research team was comprised of individuals of varied backgrounds. Researcher K.D. (female; she/her/hers) is a trained medical education specialist who does not work clinically or directly with internal medicine faculty or trainees and was thus selected to lead the focus groups. Researcher M.M. (female; she/her/hers) is a clinician who attends on the inpatient resident teams. M.K. (male; he/him/his) is a resident in the Massachusetts General Hospital (MGH) program, and A.S.B. (female; she/her/hers) is a trained clinician who now works exclusively in educational research.

#### Relationship with participants

Researchers discussed and reflected on how M.M., M.K., and A.S.B. belonging to the role groups targeted in the focus group provided insight and context into the statements made by residents and attendings but represented an area that could be influenced by preconceived notions and bias.

### Study design

#### Theoretical framework

The content analysis underpins this study. We conducted a qualitative study through 5 semi-structured focus group interviews of resident and attending physicians. Qualitative methodology is presented in accordance with the COREQ (Consolidated Criteria for Reporting Qualitative Research) checklist.

#### Participant selection

We conducted focus groups of residents and attendings in the Department of Medicine at MGH, a large, urban, US academic medical center from December 2018 to March 2019. The first focus group—including internal medicine and pediatric chief residents—was used as a pilot to assess study feasibility and facilitator guide efficacy. We held 2 additional attending and 2 resident focus groups. Recruitment occurred via email. Participants received a US $20 gift card to the hospital coffee shop and a meal as compensation.

Participants were drawn from 3 groups: chief residents, residents, and faculty. Chief residents at our institution have completed residency and stay on for a fourth post-graduate year to serve as residency administrators, leaders, and attending physicians on inpatient medical wards supervising residents. Thus, the chief resident focus group was considered an attending group in the study.

Internal medicine residents at our institution complete a 3-year residency guided by ACGME requirements. Focus group participants were recruited from those participating in the “Resident as Teacher” elective, a 2-week elective rotation for those specifically interested in improving their medical education skills. One resident focus group included first-year residents, and the second included second- and third-year residents.

The first attending focus group was comprised of core educator faculty (CEF). Members of the CEF are hospitalists identified as medical education leaders by our institution and spend most of their clinical time on teaching services. The second attending focus group was comprised of hospitalists who were not CEF but are interested in medical education and attend on resident teaching services.

#### Data collection

Investigator K.D. conducted the 60-minute focus groups, which solely included participants and occurred at a time when participants did not have clinical responsibilities. A semi-structured facilitator guide was used to elicit participant responses. The facilitator guide was developed through study investigator consensus to ensure broad exploration of perceptions of feedback and teaching in the clinical setting (Supplement 1). It was comprised of a series of open-ended questions related to teaching, feedback, and differentiating them in practice. Participants were asked to identify examples of feedback and teaching, both times they occurred together and times they occurred separately. To explore why residents might have difficulty recognizing feedback when it occurs with teaching, these questions were followed by an exercise asking participants to compare 2 scenarios. The scenarios were identified by residents and faculty in our prior study as one of stand-alone teaching and a second of both feedback and teaching [7]. Focus groups were not repeated.

The investigators met after the pilot and first attending and resident groups to update the facilitator guide with 2 additional questions based on early emerging themes. No questions were altered or removed. Focus groups were held in a private conference room, audiotaped, and transcribed verbatim by a professional transcription service. Participants were not given access to the transcripts or subsequent codes and results. All transcripts were de-identified prior to analysis. K.D. took field notes of focus groups and compared them to the transcribed interviews for accuracy.

### Data analysis

Transcripts were analyzed using thematic analysis to identify themes in participant perceptions of feedback and teaching [9]. Two investigators (M.M., M.K.) used open coding to independently generate lists of preliminary codes based on review of the pilot, 1 attending, and 1 resident transcript. They subsequently met to reflect on the analysis and compare, refine, and reconcile the codes. When the 2 investigators were unable to reach a consensus, transcripts and codes were brought to the larger group of investigators (K.D., A.S.B.) to reconcile. Two separate codebooks were generated, 1 for attendings and 1 for residents.

The 2 investigators then independently applied the codebooks to the remaining attending and first-year resident transcripts, respectively, adding additional codes where appropriate. The coders then again compared, collapsed, and refined the codes into 2 final codebooks with detailed definitions and examples of each code. We found only minor discrepancies and reached consensus by discussion with the larger group of investigators. NVivo ver. 12.6.0 (QSR International Pty. Ltd., Doncaster, VIC, Australia) was used to organize and retrieve coded data. Themes were identified by reviewing the codes and meeting as a team to discuss, clarify, and rename themes until all codes were categorized and the team agreed thematic saturation was reached. The final author (A.S.B.) reviewed a sample of quotes under each theme to check for their trustworthiness.

## Results

The 5 focus groups included 3 attending (n=20) and 2 resident (n=13) groups. The analysis identified 71 unique codes, including 39 codes in both the attending and resident focus groups, 16 codes only in the attending focus groups, and 16 codes only in the resident focus groups. From the 71 codes, 21 categories (subthemes) were identified, from which 7 central themes were derived: feedback confusion; reactive feedback and proactive teaching; resident vulnerability; nature of the relationship; explicit signposting of feedback; delivery style and setting; and feedback in the group setting (Table 1).

These coalesced into 3 thematic categories: (1) themes that provided new insights into our specific research question to disentangle feedback and teaching (feedback confusion; reactive feedback and proactive teaching); (2) themes that highlight the importance of high-quality delivery for feedback identification (resident vulnerability; nature of relationship; explicit signposting of feedback; delivery style and setting); and, (3) an additional theme that provided insight on feedback in the group setting. Themes and thematic categories are described below utilizing quotations from residents and attendings, labeled R1-13 and A1-20, respectively.

### Thematic category 1: disentangling feedback and teaching

Participants highlighted that both residents and attendings are sometimes confused about what feedback is and how it differs from teaching, which makes feedback identification in clinical learning more difficult. Both residents and attendings defined specific ways to increase clarity by delineating feedback and teaching.

#### Feedback confusion

Participants proposed that residents struggle to identify feedback because they are confused about what constitutes feedback. They considered that this may stem from the reality that multiple types of feedback (i.e., formative and summative) are labeled generically as feedback and that feedback is ubiquitous alongside teaching in the clinical learning environment and thus often hard to explicitly pinpoint. Drawing the line between what is teaching, what is feedback, and what is something else entirely becomes difficult for residents and even attendings.

“Isn’t it all nuggets of wisdom? What people tell us, whether it’s advice in life or medical knowledge-based stuff. I don’t know. I think it’s all, it’s that, I have a hard time separating teaching and feedback.” (R5)
“If you’re an attending on the wards, when are you teaching? Always is the answer. And I think the same might go for feedback as well...It certainly makes it harder for me to know when and how I’m getting feedback, and so I assume it makes it harder for the learners to know when and how I’m [giving] feedback.” (A10)

By highlighting the significant confusion surrounding what defines feedback in clinical learning, participants demonstrated the need for clearer guidance on what should be considered feedback and how it is distinct from teaching in clinical learning.

#### Reactive feedback and proactive teaching

Respondents perceived feedback as reactive and focused on correcting past behavior and actions, differing from teaching, which was perceived as proactive and preemptive. Therefore, one potential solution participants identified to improve the confusion surrounding feedback in clinical learning is to clearly delineate feedback from teaching with regards to the time frame of the behavior or action. Where feedback is reactive; teaching is proactive.

“I was just teaching you about some other things, complications that could come [up]. And that’s showing you that it’s actually for the future [so it’s teaching]. Whereas, if you get them to tease apart what they were looking at for the past, what you’ve done versus what’s more just guiding you into the future [that’s feedback].” (R5)
“The biggest difference is the time frame. In the first scenario, the information is given after a behavior or after an action [so it’s feedback]. The second scenario, it’s implied that it’s given before an action is taken [so it’s teaching].” (A15)

### Thematic category 2: delivering high-quality feedback

Residents and attendings frequently discussed high-quality feedback as integral to improving feedback identification.

#### Resident vulnerability

Residents and attendings recognized that residents are in a vulnerable position. This can lead attendings to blunt their feedback and hesitate to signal that they are providing feedback so as not to make residents feel criticized.

“We’re all putting so much into this job and giving up a lot to do it...I think we’re quite sensitive about our performance.” (R7)

#### Nature of relationship

Participants noted the importance of the relationship between a resident and attending in facilitating effective feedback. In particular, trust was explicitly highlighted as key to aid in feedback identification.

“Trust...between team members is a very important factor, and I think it’s not common, but once in a while you’ll...have an attending or a senior resident that, where I either don’t trust their opinion fully or we don’t have a great relationship, and then it’s harder to tune in all the time to everything that they’re saying.” (R4)

#### Explicit signposting of feedback

Participants identified the importance of using specific language to explicitly identify feedback, such as signposting. Given that feedback is given regularly in clinical teams, participants noted the significance of educating teams and residency programs to create a culture of feedback. This culture should foster an understanding of what feedback is, explain how to identify it, and highlight when to expect it, even if the word feedback is not explicitly used.

“I’ve always found that the best feedback is labeled and is identified as clearly as feedback as is possible. Like saying, “[NAME], I’m going to give you some feedback on that,” using the word feedback.” (A1)
  “[It would help to preface] a block of whatever rotation you’re on by saying, ‘We’re going to do feedback in different ways: some of it is going to be on the fly as we’re going on rounds and some of it’s going to be sitting down, but I consider all of that feedback that I’m giving to you.” (A6)

In contrast to the well-studied practice of signposting, participants found it difficult to identify feedback when impersonal language is used to deliver the feedback.

“The use of second person, which we do all of the time, ‘we typically don’t do this. We want to know,’ I think could be confusing.” (A15)

#### Delivery style and setting

Participants frequently indicated that how feedback is delivered is critical to feedback identification. This includes preparing adequately, using a private setting, and using in-the-moment feedback.

“I think it’s similar to having a family meeting where you have a setup before you even have a discussion. You need to be in the room, the correct seating arrangement. You need to have a setup.” (R4)

### Thematic category 3: feedback in the group setting

Initial focus groups identified providing feedback in a group as a unique challenge of clinical feedback. As such, later focus groups were asked to comment on group feedback specifically. Participants reported that formative feedback is difficult to give and consequently rarely given in a group setting due to concerns that it may negatively impact the team dynamic. The exception is specific opportunities to highlight something a resident did well in front of a group, which can be done to increase positive feedback and behaviors on a team.

“You want to give good feedback to your team. If you say something critical, what you don’t want to do is then have that team start to point fingers, ‘Well, it’s because of so and so.’ You know what I mean? So, I think the stakes are higher to do it midstream with a clinical team that has to continue working together.” (A14)
“If you want to encourage the behavior on the team, then I think that’s a very powerful moment to be like, ‘Did you guys see that? He did this, she did that. To me, that shows this.’ [That] will make this happen in the future. So, it’s still sort of a powerful moment for a team” (A2)

## Discussion

### Key results

Our analysis suggests that delineating feedback from teaching with regards to the time frame of the behavior or action is helpful: feedback focuses on a response to a behavior or action (reactive), while teaching focuses on an approach before an action is taken (proactive). Importantly, residents and attendings had significant overlap on the critical aspects of feedback delivery and identification. Our results reinforce known findings regarding feedback identification, but also provide new important insights into both the understudied phenomenon that feedback is not easily identified in the context of teaching and insight on feedback in the group setting [10,11].

### Interpretation

Previous work showed that residents have difficulty identifying and differentiating feedback and teaching in the clinical setting [7]. This analysis demonstrates one possible reason for this difficulty is that feedback and teaching are ubiquitous in the clinical learning environment, creating confusion in their distinction. Feedback and teaching each hold their own importance and ensuring they can be explicitly separated and identified is important. To progress in their development, learners require data on their performance, self-reflection on their performance, and processing capacity to integrate data and reflections into future performance [6,12, 13].

Knowing when the data being provided is feedback and when it is teaching would improve a learner’s processing capacity, allowing for better understanding and contextualization of the new information. Given the increasing emphasis on residents’ ability to learn and grow via feedback [4], it would seem critical that residents explicitly understand the differences between feedback and teaching. While feedback is reactive—responding to past actions—it also plays an important role in helping translate feedback into future growth and learning.

This study re-emphasizes prior work demonstrating that learners feel vulnerable receiving feedback [10]. The reflective component of feedback may be partly responsible for this vulnerability, as it asks learners to look back at past achievements and mistakes. Residents and attendings identified the importance of the reflective aspect of feedback in the theme “reactive feedback and proactive teaching.” They differentiated teaching by noting that it does not require this same retrospective component and instead guides actions that have not yet taken place. Thus, teaching may serve its own important and separate role in increasing knowledge while requiring far less vulnerability, possibly improving psychological safety. Psychological safety is critical for high-functioning clinical teams, fostering relationship-building and allowing learners to “focus on learning in the present moment without considering the consequences for their image in the eyes of others [14].” Residents frequently have difficulty identifying feedback in clinical environments for factors including the relationship between a learner and supervisor, the learner’s mindset, and the culture of feedback in the learning environment [5,6], which tie into psychological safety.

As a proactive endeavor that does not require reflection, teaching may require a lower threshold of psychological safety for learners to internalize. Learning via teaching does not require the strong feedback culture, precise language, and high-quality relationships that residents need to feel psychologically safe enough to internalize feedback. Teaching brings an important, unique component to the learning environment that requires less vulnerability; however, it misses the critical aspects of self-reflection. As feedback is vital to learners’ growth, ongoing efforts to improve feedback identification through resident and faculty education should be prioritized.

Effectively utilizing group feedback may help create a culture of psychological safety. Although there are challenges associated with formative group feedback, participants noted the benefit of highlighting what a resident did well in group settings. Group debriefs are commonly utilized in simulation and can improve clinical outcomes [15]. Further research is warranted to explore whether incorporating positive feedback in a group setting decreases vulnerability and increases psychological safety.

### Limitations

Potential bias related to reflexivity is possible. The study authors were aware of the prior study’s work demonstrating difficulty with the overlap of feedback and teaching. Additionally, as residents and attendings themselves, some authors likely had preconceived notions about the difference between feedback and teaching. Participants represented residents and attendings in only one program. However, MGH has a large residency program with diverse views that likely represent a variety of perspectives. We specifically included attendings and residents with a demonstrated interest in medical education. While this likely increased our engagement and breadth of responses, feedback is not solely delivered and received by residents and faculty with medical education experience. Therefore, our responses may not reflect the broader community of residents and attendings.

### Suggestions

Attendings and residents should acknowledge confusion surrounding what defines feedback in clinical learning and seek to explicitly define when feedback versus teaching is given. By considering feedback as reactive and teaching as proactive, together with effective and explicit delivery of feedback, confusion between the two may be minimized and learning potentially increased. Encouraging feedback in group settings may help to create a psychologically safe learning environment.

### Conclusions

Overall, residents and attendings highlighted important themes in discriminating feedback from teaching to aid its identification in the clinical setting. Researchers should prioritize work to improve and educate around explicit definitions of feedback and how to deliver it effectively and in a safe learning environment.

## Figures and Tables

**Table 1.** Resident and attending codes by category

Theme	Code
Disentangling feedback and teaching	
Feedback and teaching overlap	Feedback and teaching are linked
	Feedback and teaching are provided together
	Good feedback is supplemented with teaching
	Teaching obscures feedback
Multiple feedback definitions exist	Multiple kinds of feedback exist
	Preconceived beliefs related to teaching and feedback^a)^
	Summative feedback is useful^b)^
Teaching is proactive and feedback is reactive	Clinical context absent indicates teaching
	Modeling as teaching^a)^
	Feedback enhanced in response to clinical decision
	Feedback should be based on observation^b)^
	Teaching is proactive and feedback is reactive
	Teaching as transaction
Delivering high-quality feedback	
Connection with learners	Clinical medicine is a revolving door^b)^
	Trust helps feedback
Feedback involves judgement	Evaluation is perceived as feedback
	Feedback compares to gold standard^b)^
	Feedback involves subjectivity
Feedback requires effort	Feedback is time intensive^b)^
	Feedback requires preparation^b)^
Feedback should be specific	Course correction is desired
	Discrete actionables are useful
	Examples are helpful
	Feedback should be limited in scope^b)^
	Generic feedback is not useful^b)^
Feedback timing matters	Just-in-time feedback is difficult to remember^a)^
	Timeliness of feedback is important
Givers recognize learner vulnerability	Blunted feedback is not clear^a)^
	Feedback is about decision not person^a)^
	Feedback recipient is vulnerable
	Signposting can be uncomfortable^a)^
Hierarchy is present	Bidirectional feedback is ideal
	Hard to give bidirectional feedback^a)^
	Peer feedback is too close in hierarchy^b)^
	Supervisor feedback identified as teaching
Learners are also responsible for feedback	Learner-initiated feedback viewed as feedback
	Learners must ask for feedback^b)^
	Reflection can encourage feedback^b)^
	Residents should take ownership
Learners feel vulnerable	Constructive feedback easier to identify^a)^
	Critical interactions not viewed as feedback
	Feedback balance appreciated^b)^
	Feedback enhances self-worth^b)^
	Feedback permission desired^b)^
	Feedback recipients feel sensitive
	Positive feedback harder to identify
Location of feedback matters	Feedback given with patients is disempowering^a)^
	Private feedback is ideal
Naming feedback is important	Closed loop feedback is useful
	Retrospective examples of feedback are helpful^b)^
	Signposting is desired
	Spaced feedback improves retention^a)^
Nonverbal communication	Feedback can include nonverbal communication^a)^
Setting expectations is key	Normalize feedback^b)^
	Set feedback expectations as a team
	Set feedback expectations in medical education
Understanding the learner is important	Discrepancy in understanding between attending and learner^a)^
	Know your learner
	Learner goal-setting is useful
	Learner state of mind is important for feedback recognition
	Understand where the learner is coming from
“You” versus “we” feedback	Feedback hard to identify when “we” used
	Impersonal feedback is not recognized^a)^
	Personal feedback is recognized
Feedback in the group setting	
Challenges of group feedback	Constructive feedback is more challenging to provide
	Constructive feedback is rarely given in a group
Challenging to define feedback	Feedback is everywhere
	Feedback is hard to define^a)^
	Labels are unnecessary
Group feedback has utility	Group feedback is useful^b)^
	Reinforce positive behavior through group feedback^a)^

All other codes found in both resident and attending codebooks.

a) Code found only in attending codebook.

b) Code found only in resident codebook.

## Supplementary materials

Data files are available from Harvard Dataverse: https://doi.org/10.7910/DVN/CE7YJS

Supplement 1. Facilitator guide for focus groups.

Supplement 2. Audio recording of the abstract.
