# Supplementary material for: Medical residents and attending physicians’ perceptions of feedback and teaching in the United States: a qualitative study
Source: J Educ Eval Health Prof. 2022 Apr 26;19:9. doi: 10.3352/jeehp.2022.19.9 (PMC9247714; doi:10.3352/jeehp.2022.19.9)
Supplement: Supplementary file 1 — Supplement 1. Facilitator guide for focus groups. [file jeehp-19-09-suppl.pdf]

Supplement 1. Facilitator guide for focus groups

Welcome and thank you for volunteering to take part in this focus group. You have been asked to participate in this focus group as your point of view is important. I realize you are busy, and I sincerely appreciate your time. Please enjoy the food and feel free to get up and get more food during the session.

Introduction

This focus group is designed to better understand your thoughts on why residents have difficulty identifying feedback in clinical settings.

Several studies across many specialties have shown that there is a disconnect between faculty and learners regarding the provision of feedback, as faculty often feel they are providing it, but learners frequently do not feel they are receiving it.

In our prior work, we surveyed residents to better understand this discrepancy and found that residents have trouble identifying feedback in the context of teaching. We want to explore this notion further, and so this focus group is meant to obtain a deeper understanding of:

1. Why residents have difficulty observing feedback in the context of teaching
2. What we can do to address this gap in identification

While much of what we will talk about today is in regard to inpatient, internal medicine feedback and teaching, any experiences that you have had may be useful to consider.

The focus group discussion will take between 45 and 60 minutes. I will be facilitating this discussion while [insert name of observer] will be taking anonymous notes. I am a researcher on the study, and I do not work in

the Department of Medicine. No one in the Department of Medicine will be aware of whether you participated or not.

Everything said in this focus group will be audiotaped and then transcribed in a de-identified way so that your opinions remain anonymous. Out of respect for your peers, please do not discuss any comments outside of this setting. No one from the Department of Medicine will hear the audio file. The transcripts will be used to inform research as well as curriculum improvement.

I'm going to turn on the recorder now, if it's ok with the group (look for group consent, if consent given, switch on the recorder).

Ground rules

- The most important rule is that only one person speaks at a time. There may be a temptation to jump in when someone is talking, but please wait until they have finished
- There are no right or wrong answers
- You do not have to speak in any particular order
- When you do have something to say, please do so. There are many of you in the group, and it is important that each of you has a chance to speak
- You do not have to agree with the views of other people in the group
- We are very interested in hearing from everyone. Before we begin, does anyone have any questions about the study?

First, I'd like everyone in the group today to introduce themselves. Can you please tell us your name and your [year in residency/how many years you have been in practice]?

1. To get started, what does the term "feedback" mean to you?

a. If needed, ask probes like:

- Why do you think that?
- Can you tell me more about that?

2. What is an example of good feedback you have seen or received on the wards?

a. If needed, ask probes like:

- Why do you think that?
- Can you tell me more about that?

3. What is an example of a time you received group or team feedback?

a. How valuable do you find feedback when it's given to a group or team?

b. If needed, ask probes like:

- What did you take away?

4. **(skip/speed through): Give an example of a time on the wards that you received [or provided] teaching without feedback

a. If needed, ask probes like:

- Why do you think that it was just teaching?
- Can you tell me more about what made it only teaching?

5. Give an example of a time that you received [or provided] feedback and teaching together

a. If needed, ask probes like:

- Why do you think that it was both teaching and feedback?
- Can you tell me more about what made it both teaching and feedback?

I am going to hand out two scenarios (paper with scenarios passed out). Experts have identified the scenario on the left as both feedback and teaching and the scenario on the right as only teaching. Please take a moment to review and compare the two scenarios. Feel free to take notes or underline parts of the scenarios that you think are key to identifying them as both feedback and teaching and only teaching, respectively. To be very clear, the one on the left is both feedback and teaching, and the one on the right is only teaching.

During a night shift, an intern wrote an order for a patient to receive nitroglycerin as needed for chest pain. After the presentation on rounds the next morning, the supervising resident said to the intern: "We typically don't write patients for PRN nitroglycerin while they are in the hospital, because we want to know about it if patients are having chest pain and evaluate them prior to administering treatment."	A supervising resident said to the team: "We typically don't write patients for PRN nitroglycerin while they are in the hospital, because we want to know about it if patients are having chest pain and evaluate them prior to administering treatment."

1. What components of each scenario allow you to identify them as teaching?

a. If needed, ask probes like:

- Why do you think that?
- Can you tell me more about that?

2. What components of the scenario on the left signal to you that it also is feedback (in addition to teaching)?

a. If needed, ask probes like:

- Why do you think that?
- Can you tell me more about that?

3. Compare the scenario on the left to the one on the right. What is the scenario on the right missing that demonstrates to you that it is not feedback?

a. If needed, ask probes like:

- Why do you think that?
- Can you tell me more about that?

4. Why do you think residents have difficulty identifying feedback in the scenario on the left?

a. If needed, ask probes like:

- Why do you think that?
- Can you tell me more about that?

5. How might we aid residents in identifying the scenario on the left as feedback?

a. Is there a way to signal to residents in the moment on rounds that this is feedback without saying "I'm going to give you some feedback now"?

b. Is there a way to educate residents more globally to be able to identify that this is feedback?

c. If needed, ask probes like:

- Why do you think that?
- Can you tell me more about that?

6. One theme that emerged from our other focus groups was the idea that feedback is reactive, while teaching is proactive. Can you tell me your thoughts about that?

a. Why do you think that?

b. Can you tell me more about that?

7. Is there anything else anyone would like to tell me about these scenarios or resident identification of feedback in general?

8. Does anyone have questions for me about the study?

I want to thank all of you again very much for your time. We appreciate your thoughtful answers and reflection a great deal.
